# Efficacy of raltegravir in achieving virological suppression at delivery in HIV-positive pregnant women: a systematic review and meta-analysis

**DOI:** 10.1186/s12884-025-08135-5

**Published:** 2025-11-26

**Authors:** Ying Zhang, Wenlin Wu, Mengru Yang, Jie Liu, Huanmei Wu, Yaokai Chen

**Affiliations:** 1 Department of Infectious Diseases, Chongqing Public Health Medical Center, 109 Baoyu Road, Shapingba, Chongqing, 400036 China; 2 Value & Implementation, Global Medical & Scientific Affairs, MSD China, Shanghai, 200233 China

**Keywords:** Human immunodeficiency virus, Raltegravir, Pregnant women, Efficacy

## Abstract

**Background:**

The world’s first HIV integrase inhibitor, raltegravir (RAL), has been widely used for the treatment of HIV infection over the past 15 years, and continues benefiting people living with HIV (PLWH) today due to its efficient virological suppression and favourable safety profile. However, to date, there have not been any published systematic analyses evaluating the efficacy of RAL in HIV-positive pregnant women. Herein, our study aimed to evaluate the efficacy of raltegravir (RAL) regarding virological suppression at delivery in Human immunodeficiency virus (HIV)-positive pregnant women by systematic review and meta-analysis.

**Methods:**

In this study, we searched PubMed, EMBASE, the Cochrane Library, and Web of Science from inception until January 17th, 2023. Clinical trials and observational studies (cohort studies, case series, case reports) were included. All analyses were performed using R 4.1.1 statistical software with random effects modelling.

**Results:**

Twenty-two studies with 806 HIV-positive pregnant women were included. Pooled analysis indicated that the overall effectiveness rate for achieving virological suppression at delivery is 85% (95% confidence interval [CI]: 79% − 90%). Analysis of secondary outcomes revealed an overall efficacy of 78% (95% CI: 72% − 84%) for those with an HIV RNA load of < 1000 RNA copies/mL at delivery when RAL was initiated from the second trimester onward. Additionally, subgroup analysis showed an overall effectiveness rate of 75% (95% CI: 58% − 87%) for treatment-naïve (*n* = 29) and 76% (95% CI: 60%-86%) for treatment-experienced patients (*n* = 38).

**Conclusion:**

It concluded that RAL is effective for achieving virological suppression at delivery in HIV-positive pregnant women, and possibly contribute to the prevention of mother-to-child HIV transmission.

**Trial registration:**

This study was registered in PROSPERO, with registration number: CRD42023428508.

**Supplementary Information:**

The online version contains supplementary material available at 10.1186/s12884-025-08135-5.

## Introduction

Globally, human immunodeficiency virus (HIV) infection remains a major public health challenge, with approximately 39.9 million people living with HIV worldwide as of 2023 [1]. Among these patients, approximately 1.3 million were newly-infected and 0.63 million died due to Acquired Immune Deficiency Syndrome (AIDS) [1]. The incidence of HIV infection among transgender women has been reported to be 4.42 per 100 person-years (PYs) in China, and 1.35 per 100 PYs among men who have sex with men [2]. Between 1994 and 2019, there has been an oscillating trend with respect to the age-standardized incidence rate of AIDS in China and an increasing trend with respect to the age-standardized incidence rate of AIDS in the United States [3]. Modern antiretroviral HIV treatment has become a cornerstone of global control of the HIV pandemic. Modern antiretroviral therapy is widely used for HIV/AIDS treatment and includes different classes of drugs, such as nucleoside reverse transcriptase inhibitors (NRTIs), non-nucleoside reverse transcriptase inhibitors (NNRTIs), protease inhibitors (PIs), integrase strand transfer inhibitors (INSTIs), fusion inhibitors (FIs), and CCR5 antagonists [4].

Raltegravir (RAL), an HIV-1 integrase strand transfer inhibitor, was the first drug in its class that was commercialized for HIV therapy, and has been widely used for the treatment of AIDS [5]. In HIV-infected pregnant women, RAL plays an important role in preventing mother-to-child HIV transmission (MTCT) [6, 7]. Early and sustained viral control reduces the risk of MTCT [8]. An open-labelled multicentre study that included 22 patients, 68% of whom started RAL-containing regimens during pregnancy, reported that 86% of patients had an undetectable viral load (˂50 copies/mL) when they approached delivery, and none of the delivered children were infected with HIV [6]. Another randomized controlled trial (RCT) including 408 women found the proportion of women with a viral load of ˂200 copies/mL at or near delivery was significantly higher in the RAL group than in the efavirenz group [7], which supports the presumption that RAL is an effective option for the treatment of HIV-infected pregnant women.

In recent times, several case series have reported on the efficacy of RAL in HIV-positive pregnant women. However, these case series had small sample sizes [9, 10]. In 2016, a systematic review of 44 pregnancies, which included case reports and case series, found that there was insufficient evidence to determine the efficacy and safety of RAL in decreasing the risk of MTCT in HIV-infected pregnant women [11]. Results of another review of 278 maternal-infant pairs who received RAL during pregnancy supported the use of RAL during pregnancy in both antiretroviral-naïve and antiretroviral-experienced women [12]. However, these systematic reviews failed to calculate the efficacy rate of RAL in terms of virological suppression. Thus, the overall effectiveness of RAL use in HIV-positive pregnant women remains unclear. We have therefore conducted the present systematic review and meta-analysis to investigate the efficacy of RAL in HIV-positive pregnant women, and to provide a reliable rationale for therapeutic decision-making in this patient population.

## Materials and methods

Our systematic review was conducted in accordance with the Preferred Reporting Items for Systematic Reviews and Meta-Analyses (PRISMA) 2020 statement [13]. This study was registered in PROSPERO, and the registration number is CRD42023428508.

### Search strategy

PubMed, Excerpta Medica database (EMBASE), Cochrane Library, and Web of Science for English literature were searched from the date of the database inception to January 17th, 2023, using the key terms “HIV”, “AIDS”, “Acquired Immune Deficiency Syndrome”, “Human Immunodeficiency Virus”, “Raltegravir”, “MK 0518”, and “women”. The detailed search strategy is shown in Supplementary Material A.

### Inclusion criteria

1. HIV-positive pregnant women, with no limitations with respect to age and duration of illness,
2. Patient acceptance of antiretroviral therapy which included RAL at a dosage of 400 mg BID,
3. Eligible RCTs and observational studies, including prospective/retrospective cohort studies, case-control studies, case series, and case reports.

### Exclusion criteria

1) Publications in languages other than English,

2) Literature types such as targeted literature reviews, protocols, editorial letters, personal opinions, posters, conference abstracts, or dissertations. Since the conference abstracts typically only provide preliminary results and lack comprehensive methodological descriptions (such as detailed study design, participant characteristics, intervention measures, outcome definitions, and statistical analysis methods), making it difficult to accurately assess the risk of bias; besides, their findings have not undergone a full peer review process, therefore the conference abstracts were excluded.

### Primary outcome

The primary outcome was virological suppression at delivery, which was defined as a maternal plasma HIV RNA viral load of < 200 copies/mL at delivery for those who initiated RAL before the third trimester (< 28 weeks) or a maternal plasma HIV RNA viral load of < 1000 copies/mL at delivery for those who initiated RAL during the third trimester (≥ 28 weeks)).

### Secondary outcomes

The secondary outcomes were: (1) a maternal plasma HIV RNA viral load of < 50 copies/mL at delivery for those who initiated RAL before the third trimester (< 28 weeks), and (2) a maternal plasma HIV RNA viral load of < 1000 copies/mL at delivery for those who initiated RAL during the second trimester (≥ 14 weeks).

### Study selection and data extraction

Two qualified reviewers independently screened the abstracts and full texts for eligibility. During the screening process, duplicate studies and those that did not meet the eligibility criteria were excluded. The remaining studies were screened by two independent reviewers to ensure that they met pre-specified study inclusion criteria, and disagreements were resolved by a third reviewer. A PRISMA flow diagram was prepared after the study screening. Data from each study were extracted independently by two reviewers using a standardized data extraction form. Any disagreement was resolved by discussion, with assistance from a third party if necessary. Where more specific information relating to a published investigation being assessed for potential inclusion in our study was lacking or was not clear, we contacted the study authors and requested further information.

### Quality assessment

Quality assessment of single-arm studies and experimental arm studies were evaluated using the “Quality Assessment Tool for Before-After (Pre-Post) Studies with No Control Group” developed by the National Heart, Lung, and Blood Institute (NHLBI) [14]. The Newcastle-Ottawa Scale (NOS) was applied to assess the quality of prospective/retrospective cohort studies, which were assigned a score of 0–9 [15]. RCTs were assessed individually using criteria recommended in the Cochrane Handbook for Systematic Reviews of Interventions 5.1.0 [16]. The risk assessment for bias was carried out employing the forms included in the Joanna Briggs Institute (JBI) guidelines for case series and case reports [17]. Disagreements were resolved by consensus.

### Data synthesis and analysis

A proportion meta-analysis was performed to obtain pooled incidences with 95% confidence intervals (CIs). The heterogeneity of studies was assessed using Cochran’s Q test and the I^2^ heterogeneity statistic, with a significance level of 0.10 and 75%. Based on a heterogeneous pool of studies, we chose the random-effects model for this analysis [18]. We conducted subgroup analysis, stratifying by treatment experience, including subgroup with treatment-experienced patients and subgroup with treatment-naïve patients. Sensitivity analysis was conducted by removing case series and case reports on primary endpoints. Funnel plots were used to determine whether publication bias existed by using a random-effects model. All analyses were performed using R 4.1.1. statistical software [R Core Team (2022). R: a language and environment for statistical computing. R Foundation for Statistical Computing, Vienna, Austria. URL https://www.R-project.org/].

## Results

### Results of study selection

**Fig. 1:**
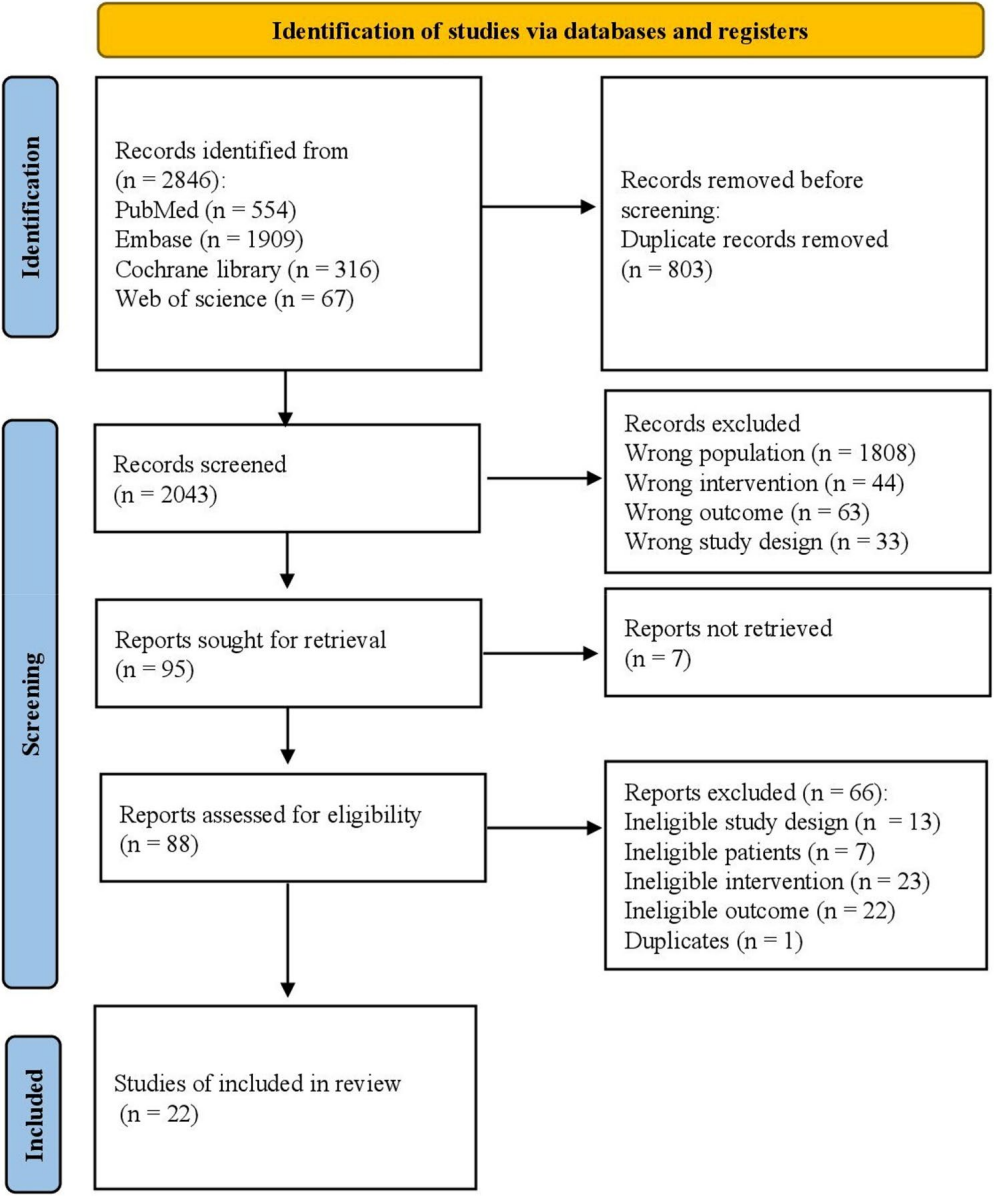
Flow diagram for the included studies

During the first screening, 2,846 studies were identified by title and abstracts through the database systematic search. After duplicates were removed, 2,043 studies remained. Following evaluation of the full texts, 88 studies remained, of which 22 were included in our meta-analysis and systematic review (Fig. 1).

### Characteristics of included studies and participants

Twenty-two [7, 9]^19^– [38] studies (2 RCTs, 7 cohort studies, 3 case series, and 10 case reports) which included 806 participants, were analyzed. Most studies were from the Americas and Europe, with a small number of studies from Africa and Asia. The median absolute CD4 + T-cell count ranged from 308 cells/mL to 448 cells/µL. The median gestational age at the start of RAL treatment ranged from 28 to 36 weeks. There were 540, 17, and 79 HIV-positive pregnant women who initiated RAL treatment at the second/third trimester, the first trimester, and before pregnancy, respectively. The median RAL exposure duration ranged from 17 days to 8.1 months. The commonly used drugs in combination with RAL were darunavir, zidovudine, lamivudine, and tenofovir (See Table 1 for details).

**Table 1:** Summary of the basic characteristics of included studies

Study ID	Study design	Sample size	Country	Region	Age (year)			Absolute CD4 count (cells per µL)			Gestational age (weeks)			Initiation time	Antiretroviral therapy	Duration of RAL
					Median	Range	Other	Median	Range	Other	Median	Range	Other			
Belissa 2015	Cohort study (no control group)	23	France	Caucasian: 2 (9%); African: 21 (91%)	31	27–38	NR	434	NR	IQR: 280–529	NR	NR	Initiation of RAL at least 2 weeks before delivery	Before pregnancy: 9 (39%) PMTCT: 3 (13%) intensification: 11 (48%)	RAL 400 mg BID+ • DRV/r 600/100 mg BID: 16 (70%) • DRV/r 800/100 mg QD: 1 (4%) • LPV/r 400/100 mg BID: 4 (17%) • SQV/r 1000/100 mg BID: 1 (4%) • FTC/TDF 200/300 mg QD: 8 (35%) • ABC/3TC 600/300 mg QD: 5 (22%) • ZDV/3TC 300/150 mg BID: 2 (8%) • ABC 600 mg QD: 3 (13%) • TDF 300 mg QD: 5 (22%)	Median (range): 8.1 (2.6–67.1) months
Boucoiran 2015	Case series	11	Canada	NR	31	21–39	NR	308 cells per mL	50–600 cells per mL	NR	35.7	31.1–38.0	NR	Third trimester	RAL 400 mg BID	Median (range): 20 (1–71) days
Brites 2018	RCT	17	Brazil	Black/Mixed	NR	> 18	Mean: 26.7	NR	NR	Mean: 451 cells per mL	NR	NR	Mean: 32.5	Third trimester	RAL 400 mg BID + co-formulated pill of zidovudine 300 mg plus lamivudine 150 mg BID	from baseline to delivery
Cecchini 2017	Retrospective cohort study	28	Argentina	Hispanic	23	NR	IQR: 19–32	300	NR	IQR: 197–436	34	29–36	NR	Third trimester	ART selection based on different clinical formulation: intensification (INS, defined as addition of RAL to current ART because of detectable antepartum viral load),13 (41.9%): FTC-TDF-ATV/r (38.5%), 3TC-AZT-LPV/r (23%), 3TC-ABC-ATV/r (7.6%), 3TC-TDF-ATV/r (7.6%), 3TC-TDF-AZT-DRV/r (7.6%), FTC-TDF-DRV/r (7.6%), 3TC-TDF-LPV/r (7.6%); late presenter (LP, (first contact with health system at > 30 week of gestational age), 15 (48.4%): 3TC-AZT-LPV/r (73.5%), FTC-TDF-LPV/r (13.3%), 3TC-TDF-LPV/r (6.6%), 3TC-TDF-ATV/r (6.6%).	Median (range): INS: 33 (29–37) days LR: 30 (7–30) days
Hoffer 2013	Case report	1	Italy	African	NR	NR	31	NR	NR	204	NR	NR	35	Third trimester	RAL 400 mg BID+(ZDV 600 mg + 3TC 300 mg) BID + LPV/r 400/200 mg BID	NR
Gantner 2019	Retrospective cohort study (no control group)	94	France	Sub-Saharan African: 80 (85%); Caucasian: 8 (9%); Maghrebin: 4 (4%); Asian: 2 (2%)	33	20–45	NR	448	4–986	NR	NR	NR	NR	Before pregnancy: 33 (35.1%) Second trimester: 11 (11.7%) Third trimester: 50 (53.2%)	RAL 400 mg BID	at least 15 days
Hegazi 2012	Case report	3	Cases 1: Rwandan; Cases 2: Ghana༛Cases 3: Uganda	NR	32	24–32	Cases 1: 24; Cases 2: 32; Cases 3: 32	NR	NR	NR	29	22–30	Cases 1: 22 Cases 2: 14 h before delivery (Gestation at delivery: 30 + 3) Cases 3: 22.5 h before delivery (Gestation at delivery: 29 + 5)	Second trimester: 1 (33.3%) Third trimester: 2 (66.7%)	Case 1: RAL + IV AZT Case 2: RAL + Nevirapine + IV AZT Case 3: RAL + Nevirapine + IV AZT	NR
Hegazi 2013	Case report	1	NR	West African	NR	NR	28	NR	NR	200	NR	NR	28	Third trimester	RAL 400 mg/d + TDF/FTC + ritonavir-boosted SQV BID	71 days
Jaworsky 2010	Case report	1	Canada	NR	NR	NR	19	NR	NR	NR	NR	NR	0	Before pregnancy	3TC/ZDV/ABC (150/300/300 mg BID), TDF (300 mg/d), etravirine (200 mg BID), DRV (600 mg BID), ritonavir (100 mg BID) and RAL (400 mg BID)	throughout pregnancy
João 2020	RCT	206	Argentina, Brazil, South Africa, Tanzania, Thailand, and the USA	Asian or Pacific Islander: 23 (11%);Black, not Hispanic: 72 (35%); Hispanic, Latino : 108 (53%);White, not Hispanic: 2 (1%)	27	23–32	NR	389.5	240–567	NR	28	22–31	20–28: 103 (50.0%) 28–37: 103 (50.0%)	Second trimester: 103 (50.0%) Third trimester: 103 (50.0%)	RAL 1400 mg BID + AZT 300 mg + 3TC 150 mg BID	NR
McKeown 2010	Case report	2	Cases 1: Ugandan; Cases 2: Zimbabwean	NR	35	31–39	Cases 1: 39 Cases 2: 31	NR	NR	NR	33.5	28–39	NR	Third trimester	Case 1: RAL + TDF/FTC, ABC etravirine Case 2: RAL + TDF/FTC, DRV/r, (IV ZDV at delivery) Case 3: RAL + TDF/FTC, efavirenz (IV ZDV at delivery)	Case 1: 11 wekks Case 2: 5 weeks
Medeiros 2016	Case series	7	Brazil	NR	NR	20–36	Mean: 26.3	NR	NR	Mean: 486	NR	21–37	Mean: 32.3	Second trimester and Third trimester	RAL 400 mg BID+ • ZDV/3TC/LPVr 4 (57.1%) • TDF/3TC/LPVr 2 (28.6%) • TDF/3TC/NVP 1 (14.3%)	NR
Nóbrega 2013	Case report	14	Brazil	NR	29.5	17–37	NR	239	65-1203	Mean: 338 cells per mL	36	34–38	NR	Third trimester	ZDV + 3TC + LPV/r + RAL 6 (42.9%) ZDV + 3TC + RAL 5 (35.7%) ZDV + 3TC + ATV/r + RAL 1 (7.1%) ZDV + 3TC + DRV/r + RAL 1 (7.1%) ZDV + 3TC + TDF + DRV/r + RAL 1 (7.1%)	Median (range): 17 (7–32) days
Patel 2022	Cohort study	86	USA	Non-Hispanic Black: 45 (52%);Non-Hispanic White or other: 14 (16%); Hispanic: 27 (31%)	NR	NR	NR	NR	NR	NR	NR	NR	NR	Before pregnancy: 45 (52.3%) First trimester: 17 (19.8%) Second trimester: 12 (14.0%) Third trimester: 12 (14.0%)	RAL	NR
Pinnetti 2010	Case report	1	Italy	NR	NR	NR	NR	NR	NR	350	NR	NR	38	Third trimester	ZDV + 3TC + DRV/R + TDF 300 mg QD + RAL 400 mg BID	9 days
Puthanakit 2018	Prospective cohort (no control group)	154	Thailand	NR	23	IQR: 19–29	NR	382 (*N* = 128)	NR	IQR: 171–545 (*N* = 128)	34	NR	IQR: 33–36	Third trimester	RAL 400 mg BID+ • TDF-3TC-EFV or TDF-FTC-EFV 66 (43%) • TDF-3TC-LPV/r 51 (33%) • ZDV-3TC-LPV/r 28 (18%) • Other regimens 9 (6%)	Median (IQR): 21 (8–34) days
Sibiude 2018	Prospective cohort study	103	France	Metropolitan France : 14 (13.6%);Sub-saharan Africa : 75 (72.8%); Other: 14 (13.6%)	NR	NR	< 25 year: 6 (5.8%); 25–34 year: 53 (51.5%); ≥35 year: 44 (42.7%)	NR	NR	<200: *N* = 2, 1.5%	NR	NR	NR	NR	RAL	NR
Soh 2015	Case report	1	UK	NR	NR	NR	33	NR	NR	70/100 cells per mL	NR	NR	33	Third trimester	combivir, DRV and ritonavir	3 weeks
Taylor 2011	Case series	5	Austria	NR	39	24–39	NR	315	188–864	NR	34	33–34	NR	Third trimester	Case 1: FTC + TDF + RAL Case 2: 3TC + LPV/r + RAL Case 3: 3TC + ZDV + LPV/r + RAL Case 4: 3TC + ZDV + LPV/r + ENF + RAL Case 5: FTC + TDF + RAL	Median (range): 23 (17–46) days
Trahan 2020	Case report	2	Canada	NR	NR	NR	NR	NR	NR	NR	NR	NR	NR	The CMIS pregnancy cohort is composed of women with HIV who are referred for management during pregnancy, at which time all HIV and obstetrical care is provided through a multidisciplinary clinic team.	Case 1: TDF/EFZ/RAL Case 2: AZT/3TC/LPV/r/RAL	NR
Watts 2014	Prospective cohort (no control group)	42	USA: 36 (86%); Brazil: 4 (10%); Argentina: 2 (5%)	Black, non-Hispanic: 22 (52%) Hispanic (regardless of race): 16 (38%); White, non-Hispanic: 3 (7%); Mixed race: 1 (2%)	30	19–43	NR	NR	NR	Second trimester: 430 (72–977), *n* = 17 Third trimester: 510 (71–1153), *n* = 41	NR	NR	< 35	Before the beginning of the 35th week of pregnancy	RAL 400 mg BID + antiretroviral therapy (no specific treatment were specified)	
Westling 2012	Case report	4	Sweden	NR	26.5	16–29	Cases 1: 25; Cases 2: 28; Cases 3: 16; Cases 4: 29	205	40–570	NR	35.5	30–37	NR	Third trimester	Case 1: TDF/FTC/LPV/r/RAL Case 2: ZDV/3TC/ATV/r/RAL Case 3: TDF/FTC/ATV/r/RAL Case 4: TDF/FTC/ATV/r/RAL	Days on raltegravir Case 1: 8 Case 2: 49 Case 3: 12 Case 4: 22

Abbreviations: *IQR* interquartile range, *NR* Not Report, *RCT * Randomized Controlled Trials, *N* Number, *RAL* raltegravir, *DRV * darunavir,* LPV* lopinavir, *SQV* saquinavir, *TDF * tenofovir, *FT*C emtricitabine, *3TC *lamivudine, *ABC* abacavir, *ZDV* zidovudine, *AZT* zidovudine, *ATV* atazanavir, *ENF* enfuvirtide, *EFZ *efavirenz, *r *ritonavir, *PMTCT* prevention of mother-to-child transmission

### Quality assessment

Both of two RCTs have low risk bias in randomisation, blinding of outcome assessor domains, allocation concealment and incomplete data domains, and have high risk bias in blinding of participants and personnel. Two retrospective non-randomized cohort studies were scored with 7 by NOS. As for quality assessment of before and after studies, most items were “Yes”, few items were assessed with “No” or “NR”. Several case reports and case series did not clearly describe the adverse events (harms) or unanticipated events, all other items were assessed as yes. The results of our quality assessment are detailed in Table**S1****-**S4.

### Primary outcomes

**Fig. 2:**
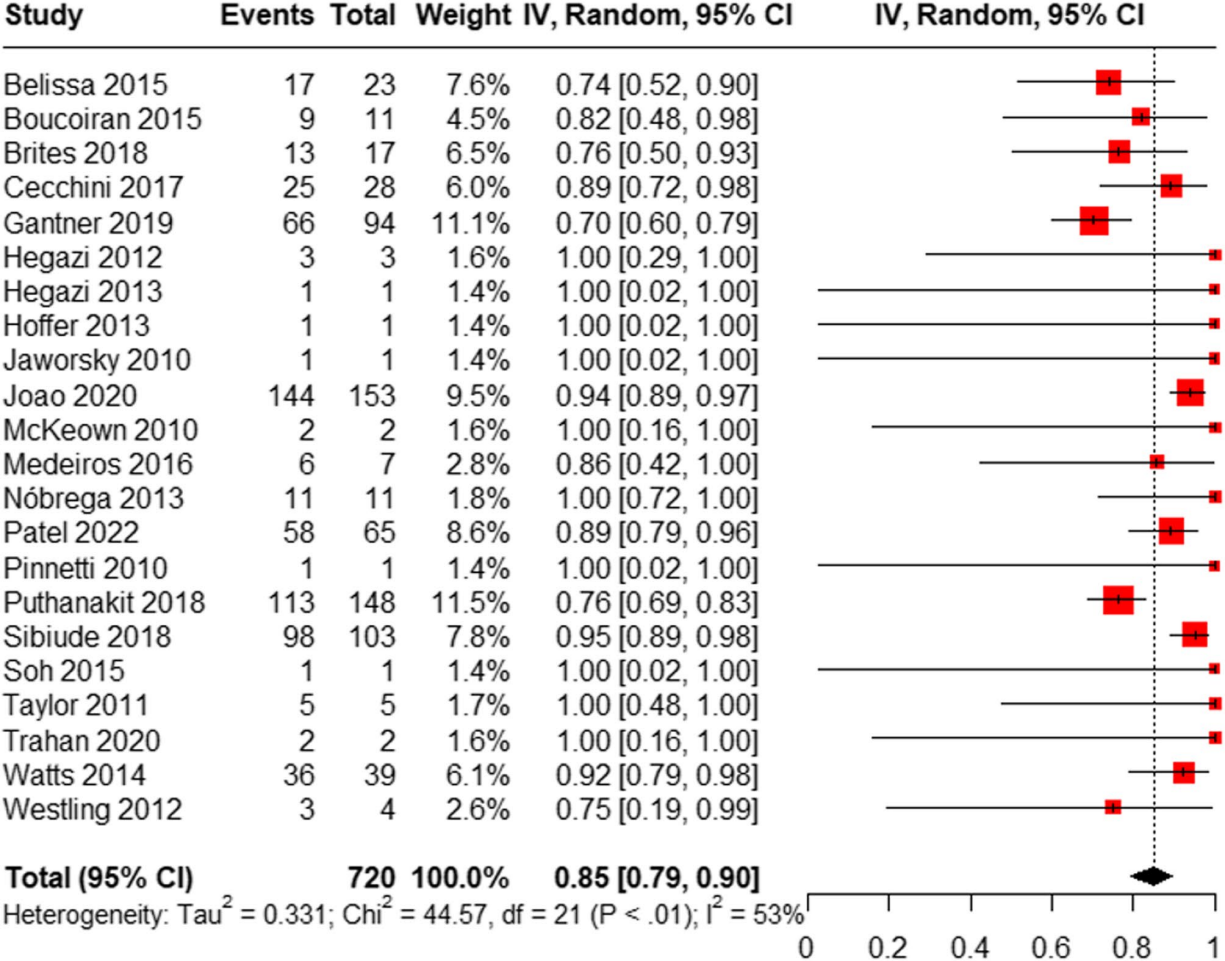
Meta-analysis forest plot of virological suppression at delivery

A total of 22 studies (including 2 RCTs, 7 cohort studies, 1 case series, and 12 case reports) involving 720 HIV-positive women reported virological suppression at delivery. Our results showed that the overall proportion of HIV-positive pregnant women with a viral load of ˂1000 copies/ml if RAL was initiated during the third trimester (≥ 28 weeks) or ˂200 copies/ml if RAL was used before the third trimester (< 28 weeks) was 85% (95% CI: [79%, 90%]) (Fig. 2).

### Secondary outcomes

**Fig. 3:**
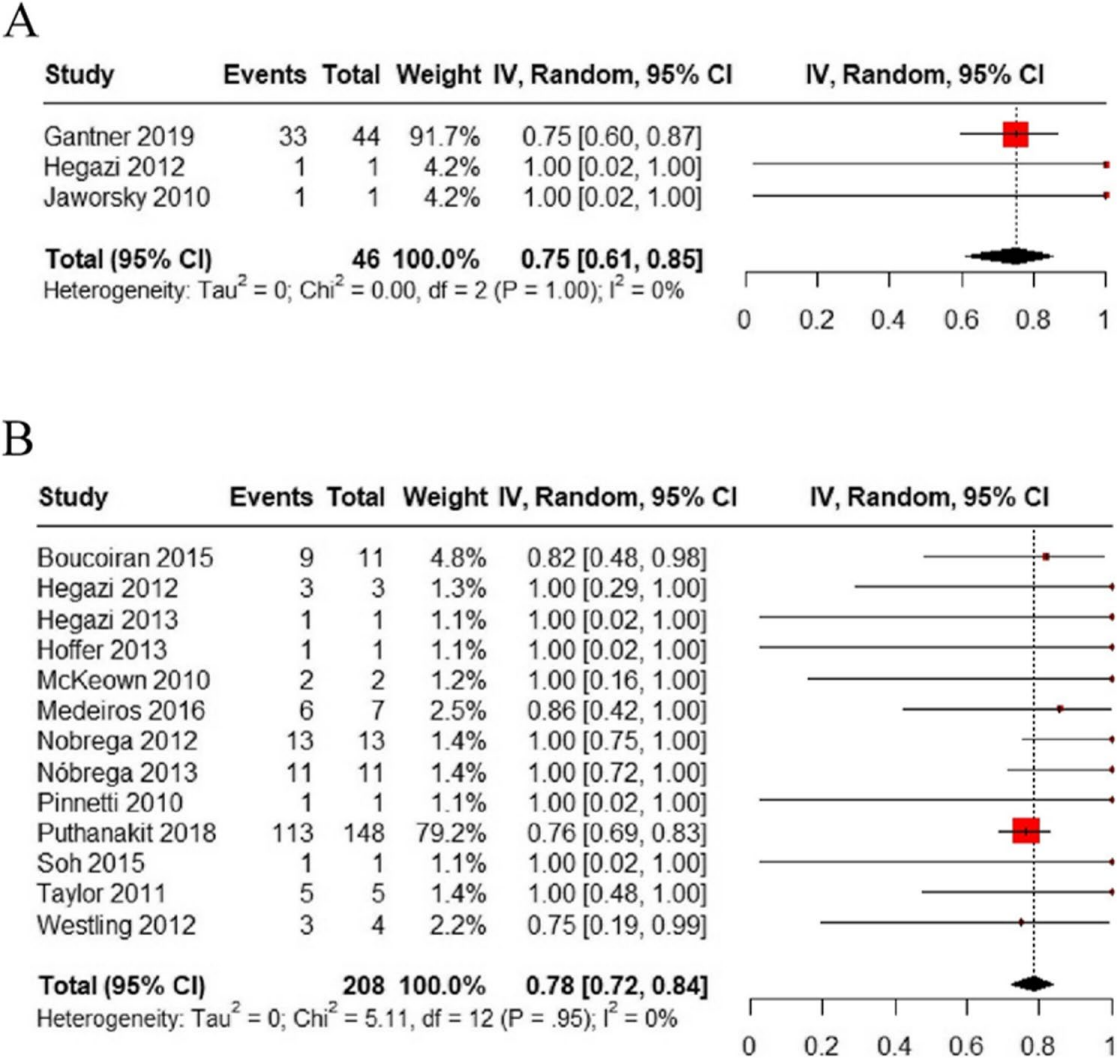
(A) Meta-analysis forest plot of virological suppression (< 50 copies/mL) at delivery if RAL was used before the third trimester (< 28 weeks); (B) Meta-analysis forest plot of virological suppression (< 1000 copies/mL) at delivery if RAL was used during the second trimester (≥ 14 weeks)

Then, we analyzed the overall proportion of pregnant women with a viral load of < 50 copies/mL at delivery if RAL was used before the third trimester (< 28 weeks). A total of 3 studies (1 cohort study and 2 case reports) with 46 HIV-positive women reported the proportion of patients with a viral load of < 50 copies/mL at delivery, and these patients received RAL before the third trimester (< 28 weeks). Our meta-analysis observed that 75% of women in this cohort achieved a viral load of < 50 copies/mL at delivery (95% CI: [61%, 85%]) (Fig. 3A).

We further analyzed the overall proportion of pregnant women with a viral load of < 1000 copies/mL at delivery if RAL was used from the second trimester (≥ 14 weeks). A total of 13 studies (3 case series, 1 cohort study, and 9 case reports) with 208 HIV-positive women reported the proportion of women with a viral load of < 1000 copies/mL at delivery and RAL was used during the second trimester (≥ 14 weeks) in these patients. Our meta-analysis observed that 78% (95% CI: [72%, 84%]) of these women achieved a viral load of < 1000 copies/mL at delivery (Fig. 3B).

### Subgroup analysis results

**Fig. 4:**
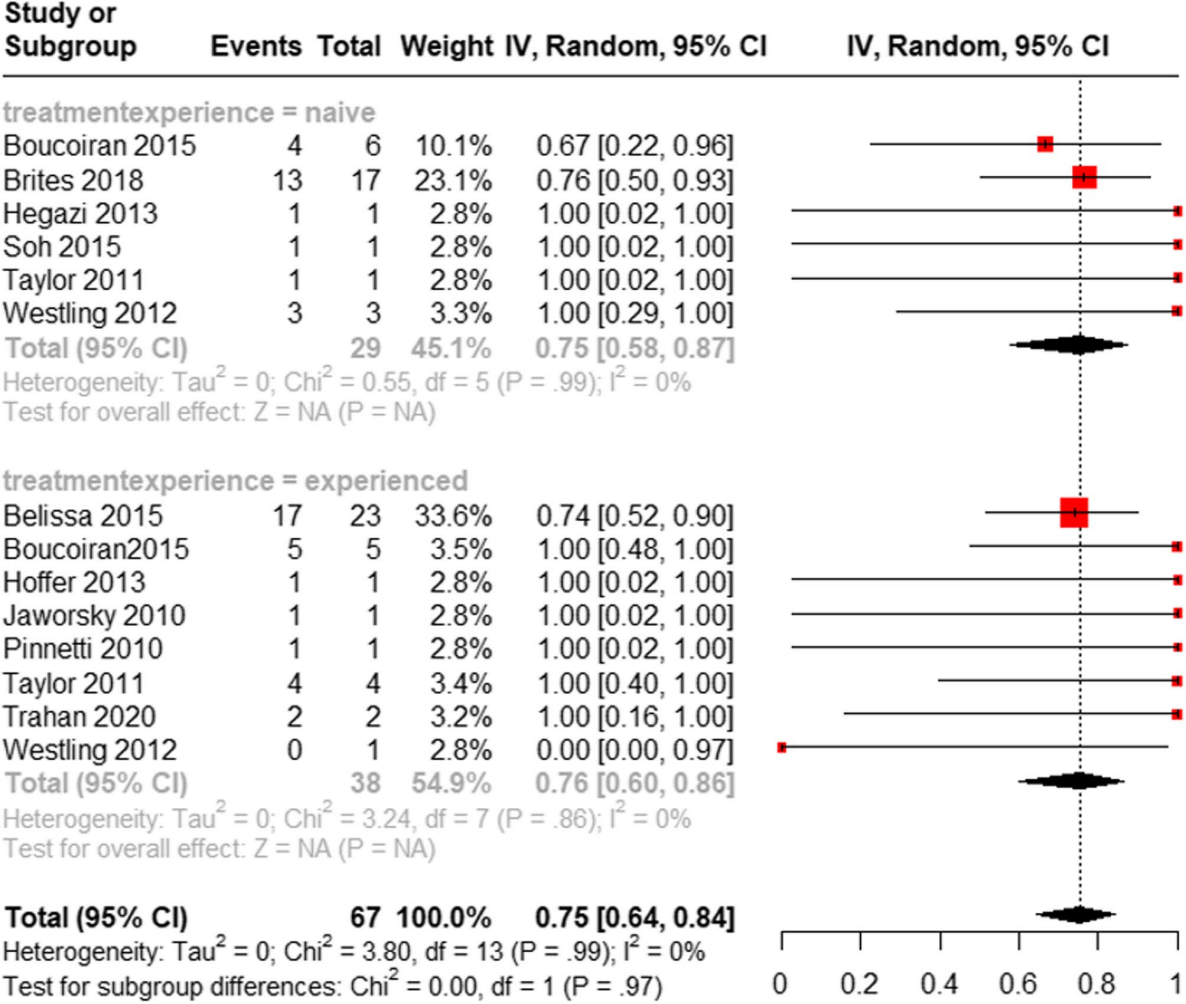
Subgroup analysis of virological suppression at delivery for treatment-naïve and treatment-experienced patients

Next, we stratified patients by treatment experience and analyzed their virological results. For treatment-naïve patients, a total of 6 studies (1 RCT, 1 case series, and 4 case reports) with 29 HIV-positive women reported the proportion of women with virological suppression at delivery, defined as a viral load of ˂1000 copies/ml if RAL was initiated during the third trimester (≥ 28 weeks) or ˂200 copies/ml if RAL was used before the third trimester (< 28 weeks). In this population, our meta-analysis showed that 75% of women who received RAL achieved viral suppression (95% CI: [58%, 87%]) (Fig. 4).

For treatment-experienced patients, a total of 8 studies (1 cohort study, 2 case series, and 5 case reports) with 38 HIV-positive women reported the proportion of women with virological suppression at delivery, defined as a viral load of ˂1000 copies/ml if RAL was initiated during the third trimester (≥ 28 weeks) or ˂200 copies/ml if RAL was used before the third trimester (< 28 weeks). Our meta-analysis observed that 76% of women in this cohort achieved viral suppression (95% CI: [60%, 86%]) (Fig. 4).

### Sensitivity analysis results and publication bias

Sensitivity analysis was conducted by removing case series and case reports. The results were consistent (**Figure ****S1**).

The funnel plot of the primary outcome appeared obviously asymmetrical, and the P value was more than 0.10, suggesting no publication bias of the meta-analysis (**Figure S2)**.

The funnel plot of the proportion of pregnant women with a viral load of < 1000 copies/mL at delivery if RAL appeared unsymmetrical and the P value is 0.0338, suggesting publication bias of the meta-analysis (**Figure S3)**.

## Discussion

To our knowledge, the present study is the first meta-analysis of directly observed efficacy rates of RAL treatment in HIV-positive pregnant women. Our results showed an overall effectiveness rate of 85% (95% CI: [79%, 90%]), with the maternal plasma HIV RNA viral load being less than 200 copies/mL at delivery in pregnant women initiating RAL-based regimens before the third trimester (< 28 weeks), and the maternal plasma HIV RNA viral load being less than 1000 copies/mL at delivery in pregnant women initiating RAL-based regimens during the third trimester (≥ 28 weeks). Our results also showed that 75% (95% CI: [61%, 85%]) of pregnant women had achieved a maternal plasma HIV RNA viral load of < 50 copies/mL at delivery when RAL was administered before the third trimester, and 78% (95% CI: [72%, 84%]) of pregnant women had achieved a maternal plasma HIV RNA viral load of < 1000 copies/mL at delivery when RAL was prescribed before the second trimester. Our subgroup analysis showed an overall effectiveness rate for viral suppression of 76% (95% CI: [60%, 86%]) and 75% (95% CI: [58%, 87%]) for treatment-naïve patients and treatment-experienced patients, respectively. Our results support the use of RAL as the preferred treatment for HIV-positive pregnant women, which is also a recommendation of the U.S. perinatal HIV transmission prevention guidelines [39].

Based on current understanding, the main determinants of perinatal HIV transmission are inadequate obstetric control, late entry into care (delayed antiretroviral therapy initiation), acute HIV infection, and non-adherence to follow-up medical appointments [21, 40]. For pregnant women living with HIV, earlier and effective antiviral treatment is essential to attain virological suppression, as the risk for mother to child transmission (MTCT) depends largely on the time of antiretroviral therapy initiation and HIV viral load before delivery [41]. One previous study reported that RAL has an acceptable safety profile and low rates of fetal toxicity when administered to pregnant women [6]. Puthanakit and colleagues reported that 76% of their study participants achieved a plasma HIV-1 RNA level of less than 1000 copies/mL at delivery, with a median reduction of 1.6 log10 RNA copies/mL during a median 21-day RAL intensification period [31]. Another study, which included 86 participants treated with RAL, observed viral suppression at delivery in 89.2% of the pregnancies, and acceptable safety profiles during pregnancy. One retrospective study in France observed that viral load was < 50 copies/mL at delivery in 82%, 55%, and 56% of women when RAL was started before pregnancy, during the second trimester, or during the third trimester, respectively [22]. Another study showed that 50% of women achieved a viral load of < 1000 copies/mL in HIV-infected women who received RAL at a median gestational age of 35.7 weeks [9]. A single-center, randomized trial conducted in Brazil, which included 33 HIV-positive pregnant women, reported that 13 of 17 (76.5%) treatment-naïve patients in the RAL group achieved virological suppression at delivery compared with 4 of 16 patients (25.0%) in the lopinavir/ritonavir group [20]. Another study of 23 treatment-experienced patients reported a virological suppression effectiveness of 61.8% at delivery with RAL [19]. Thus, a significantly higher proportion of both treatment-naïve and treatment-experienced patients achieved viral suppression at delivery in the RAL group than in other treatment groups. Furthermore, although RAL has been observed to be extensively variable in its pharmacokinetic profile, its effectiveness during pregnancy remains stable [6, 36]. The preceding observations, together with the findings of our meta-analysis, endorse the use of RAL to expedite HIV virological suppression in pregnant women who have HIV infection, even though they may have a high virological load or a suboptimal degree of virological suppression late in pregnancy when initiating RAL-containing regimens [29].

We acknowledge that there are several limitations to our study. First, among patients with HIV-infection, pregnant women belong to a special patient group that is hardly involved in large clinical trials, and most of the studies are case reports or case series with small sample sizes. Nonetheless, we had systematically collected all relevant evidence, including a larger number of case reports and case series. To ensure that the analysis results are credible, we did a sensitivity analysis by excluding these studies, but the analysis was highly heterogeneous. Therefore, high quality clinical trials are needed in the future to further validate this. Second, although our study pooled the results of all published studies, our total sample size remained relatively small. Third, most studies included in our meta-analysis were retrospective in nature and were of relatively poor quality. However, despite the above limitations, we remain convinced that our study has provided robust evidence for the efficacy of RAL use in HIV-positive pregnant women.

## Conclusion

Our results have shown that RAL is effective in achieving virological suppression at delivery in HIV-positive pregnant women, and is likely to also contribute to the prevention of mother-to-child HIV transmission.

## Supplementary Information

Supplementary Material 1.

## Data Availability

No datasets were generated or analysed during the current study.

## Abbreviations

AIDS: Acquired Immune Deficiency Syndrome
CIs: confidence intervals
Fis: fusion inhibitors
HIV: human immunodeficiency virus
INSTIs: integrase strand transfer inhibitors
JBI: Joanna Briggs Institute
MTCT: mother-to-child HIV transmission
NRTIs: nucleoside reverse transcriptase inhibitors
NNRTIs: non-nucleoside reverse transcriptase inhibitors
NHLBI: National Heart, Lung, and Blood Institute
NOS: The Newcastle-Ottawa Scale
PIs: protease inhibitors
PRISMA: Preferred Reporting Items for Systematic Reviews and Meta-Analyses
RAL: Raltegravir
RCT: randomized controlled trial
