# Supplementary material for: Efficacy of raltegravir in achieving virological suppression at delivery in HIV-positive pregnant women: a systematic review and meta-analysis
Source: BMC Pregnancy Childbirth. 2025 Nov 26;26:63. doi: 10.1186/s12884-025-08135-5 (PMC12821861; doi:10.1186/s12884-025-08135-5)
Supplement: Supplementary file 1 — Supplementary Material 1. [file 12884_2025_8135_MOESM1_ESM.docx]

**Supplementary Material A**

**Search strategy**

**PubMed**

(("HIV"[mesh] OR "Acquired Immunodeficiency Syndrome"[mesh] OR "Human Immunodeficiency Virus*"[tw] OR "LAV-HTLV-III"[tw] OR "Lymphadenopathy-Associated Virus*"[tw] OR "Acquired Immune Deficiency Syndrome Virus*"[tw] OR HIV[tw] OR AIDS[tw] OR "Acquired Immune Deficiency Syndrome*"[tw] OR "Acquired Immunodeficiency Syndrome*"[tw]) AND ("Raltegravir Potassium"[mesh] OR Raltegravir[tw] OR "MK 0518"[tw] OR MK0518[tw] OR Isentress[tw] OR RAL[tw]) AND ("Female"[mesh] OR female*[tw] OR woman[tw] OR women[tw]) AND ("Young Adult"[mesh] OR "Middle Aged"[mesh] OR "Adult"[Mesh:NoExp] OR pregnan*[tw] OR mother-to-child[tw] OR childbearing[tw]) AND ("controlled clinical trial"[pt] OR "Controlled Clinical Trials as Topic"[mesh] OR "Random Allocation"[mesh] OR "Double-Blind Method"[mesh] OR "single-blind method"[mesh] OR "Control Groups"[mesh] OR "cross-over studies"[mesh] OR random*[tiab] OR placebo[tiab] OR trial[tiab] OR groups[tiab] OR crossover[tiab] OR cross-over[tiab] OR "Observational Study"[pt] OR "Case Reports"[pt] OR "Observational Studies as Topic"[mesh] OR "Cohort Studies"[mesh] OR "Case-Control Studies"[mesh] OR "Cross-Sectional Studies"[mesh] OR Observational Stud*[tiab] OR Cohort[tiab] OR "Follow-Up"[tiab] OR Longitudinal*[tiab] OR Prospectiv*[tiab] OR Retrospectiv*[tiab] OR "Case-Control"[tiab] OR "Cross-Sectional"[tiab] OR "case series"[tiab] OR "single arm"[tiab] OR "Case Report*"[tiab]) NOT ("Animals"[mesh] NOT ("Humans"[mesh] AND "Animals"[mesh])))

**EMBASE**

((exp "Human immunodeficiency virus"/ OR exp "Acquired Immune deficiency Syndrome"/ OR "Human Immunodeficiency Virus*".mp OR "LAV-HTLV-III".mp OR "Lymphadenopathy-Associated Virus*".mp OR "Acquired Immune Deficiency Syndrome Virus*".mp OR HIV.mp OR AIDS.mp OR "Acquired Immune Deficiency Syndrome*".mp OR "Acquired Immunodeficiency Syndrome*".mp) AND ("Raltegravir"/ OR Raltegravir.mp OR "MK 0518".mp OR MK0518.mp OR Isentress.mp OR RAL.mp) AND (exp "Female"/ OR female*.mp OR woman.mp OR women.mp) AND (exp "Young Adult"/ OR exp "Middle Aged"/ OR "Adult"/ OR pregnan*.mp OR mother-to-child.mp OR childbearing.mp) AND (exp "controlled clinical trial"/ OR exp "Controlled Clinical Trial (Topic)"/ OR "Randomization"/ OR "Doubl -Blind Procedure"/ OR "single blind procedure"/ OR exp "Control Group"/ OR exp "crossover procedure"/ OR random*.ti,ab OR placebo.ti,ab OR trial.ti,ab OR groups.ti,ab OR crossover.ti,ab OR cross-over.ti,ab OR "Observational Study"/ OR "Case Report"/ OR exp "Cohort Analysis"/ OR exp "Case Control Study"/ OR exp "Cross-Sectional Study"/ OR Observational Stud*.ti,ab OR Cohort.ti,ab OR "Follow-Up".ti,ab OR Longitudinal*.ti,ab OR Prospectiv*.ti,ab OR Retrospectiv*.ti,ab OR "Case-Control".ti,ab OR "Cross-Sectional".ti,ab OR "case series".ti,ab OR "single arm".ti,ab OR "Case Report*".ti,ab) NOT (exp "Animals"/ NOT (exp "Humans"/ AND exp "Animals"/)))

**Cochrane** **Library**

(("Human immunodeficiency virus" OR "Acquired Immun deficiency Syndrome" OR "Human Immunodeficiency Virus*" OR "LAV HTLV III" OR "Lymphadenopathy Associated Virus*" OR "Acquired Immune Deficiency Syndrome Virus*" OR HIV OR AIDS OR "Acquired Immune Deficiency Syndrome*" OR "Acquired Immunodeficiency Syndrome*") AND ("Raltegravir" OR Raltegravir OR "MK 0518" OR MK0518 OR Isentress OR RAL) AND ("Female" OR female* OR woman OR women) AND ("Young Adult" OR "Middle Aged" OR "Adult" OR pregnan* OR mother to child OR childbearing)):ti,ab,kw

**Web of Science**

TS=(("Human immunodeficiency virus" OR "Acquired Immun deficiency Syndrome" OR "Human Immunodeficiency Virus*" OR "LAV-HTLV-III" OR "Lymphadenopathy-Associated Virus*" OR "Acquired Immune Deficiency Syndrome Virus*" OR HIV OR AIDS OR "Acquired Immune Deficiency Syndrome*" OR "Acquired Immunodeficiency Syndrome*") AND ("Raltegravir" OR Raltegravir OR "MK 0518" OR MK0518 OR Isentress OR RAL) AND ("Female" OR female* OR woman OR women) AND ("Young Adult" OR "Middle Aged" OR "Adult" OR pregnan* OR mother-to-child OR childbearing) AND ("controlled clinical trial" OR "Clinical Trial" OR "trial" OR "Randomization" OR "RCT" OR "Doubl -Blind" OR "single blind" OR "Control Group" OR "crossover" OR "cross over" OR random* OR placebo OR trial OR groups OR crossover OR cross-over OR "Observational Study" OR "Case Report" OR "Cohort Analysis" OR "Case Control Study" OR "Cross-Sectional Study" OR Observational Stud* OR Cohort OR "Follow-Up" OR Longitudinal* OR Prospectiv* OR Retrospectiv* OR "Case-Control" OR "Cross-Sectional" OR "case series" OR "single arm" OR "Case Report*")) NOT TI=("veterinary" OR "rabbit" OR "rabbits" OR "animal" OR "animals" OR "mouse" OR "mice" OR "rodent" OR "rodents" OR "rat" OR "rats" OR "pig" OR "pigs" OR "porcine" OR "horse" OR "horses" OR "equine" OR "cow" OR "cows" OR "bovine" OR "goat" OR "goats" OR "sheep" OR "ovine" OR "canine" OR "dog" OR "dogs" OR "feline" OR "cat" OR "cats")

**Table S1. Risk of bias for RCTs**

| **Study ID** | **Randomisation** | **Allocation concealment** | **Blinding of** **participants and personnel** | **Blinding of outcome assessor** | **Incomplete data** | **Selective reporting** | **Other bias** |
| --- | --- | --- | --- | --- | --- | --- | --- |
| Brites 2018 | Low | Low | High | Low | Low | Low | Unclear |
| João 2020 | Low | Low | High | Low | Low | Low | Unclear |

**Table S2. Risk of bias for non-randomized cohort studies**

| Study ID | | | Patel 2022 | Sibiude 2018 |
| --- | --- | --- | --- | --- |
| Selection | Representativeness of the exposed cohort | a) truly representative of the average condition in the community* ¯  b) somewhat representative of the average condition in the community* ¯  c) selected group of users eg nurses, volunteers  d) no description of the derivation of the cohort | b1 | b1 |
|  | Selection of the non-exposed cohort | a) drawn from the same community as the exposed cohort*  b) drawn from a different source  c) no description of the derivation of the non-exposed cohort | a1 | a1 |
|  | Ascertainment of exposure | a) secure record (e.g., surgical records) *  b) structured interview* ¯  c) written self-report  d) no description | a1 | d0 |
|  | Demonstration that outcome of interest was not present at start of study | a) yes* ¯  b) no | a1 | a1 |
| Comparability | Comparability of cases and controls on the basis of the design or analysis | a) study controls for age*¯  b) study controls for any additional factor* | 0 | a1b1 |
| Outcome | Assessment of outcome | a) independent blind assessment* ¯  b) record linkage* ¯  c) self-report  d) no description | b1 | d0 |
|  | Was follow-up long enough for outcomes to occur | a) yes*  b) no | a1 | a1 |
|  | Adequacy of follow up of cohorts | a) complete follow up - all subjects accounted for* ¯  b) subjects lost to follow up unlikely to introduce bias - small number lost - < 20 % follow up, or description provided of those lost )* ¯  c) follow up rate > 20% and no description of those lost  d) no statement | a1 | a1 |
|  |  | Score | 7 | 7 |

**Table S3. Risk of bias for before and after studies**

| Study ID | | Gantner 2019 | Puthanakit 2018 | Cecchini 2017 | Watts 2014 | Belissa 2015 |
| --- | --- | --- | --- | --- | --- | --- |
| 1. Was the study question or objective clearly stated? | Yes | Yes | Yes | Yes | Yes | Yes |
|  | No |  |  |  |  |  |
|  | Other  (CD, NR, NA) * |  |  |  |  |  |
| 2. Were eligibility/selection criteria for the study population prespecified and clearly described? | Yes | Yes | Yes | Yes | Yes | Yes |
|  | No |  |  |  |  |  |
|  | Other  (CD, NR, NA) * |  |  |  |  |  |
| 3. Were the participants in the study representative of those who would be eligible for the test/service/intervention in the general or clinical population of interest? | Yes | Yes | Yes | Yes | Yes | Yes |
|  | No |  |  |  |  |  |
|  | Other  (CD, NR, NA) * |  |  |  |  |  |
| 4. Were all eligible participants that met the prespecified entry criteria enrolled? | Yes | Yes | Yes | Yes | Yes | Yes |
|  | No |  |  |  |  |  |
|  | Other  (CD, NR, NA) * |  |  |  |  |  |
| 5. Was the sample size sufficiently large to provide confidence in the findings? | Yes |  |  |  | Yes |  |
|  | No | NR | NR | NR |  | NR |
|  | Other  (CD, NR, NA) * |  |  |  |  |  |
| 6. Was the test/service/intervention clearly described and delivered consistently across the study population? | Yes | Yes | Yes | Yes |  |  |
|  | No |  |  |  | No | No |
|  | Other  (CD, NR, NA) * |  |  |  |  |  |
| 7. Were the outcome measures prespecified, clearly defined, valid, reliable, and assessed consistently across all study participants? | Yes | Yes | Yes | Yes | Yes | Yes |
|  | No |  |  |  |  |  |
|  | Other  (CD, NR, NA) * |  |  |  |  |  |
| 8. Were the people assessing the outcomes blinded to the participants' exposures/interventions? | Yes |  |  |  |  |  |
|  | No |  |  |  |  |  |
|  | Other  (CD, NR, NA) * | NR | NR | NR | NR | NR |
| 9. Was the loss to follow-up after baseline 20% or less? Were those lost to follow-up accounted for in the analysis? | Yes | Yes | Yes | Yes | Yes | Yes |
|  | No |  |  |  |  |  |
|  | Other  (CD, NR, NA) * |  |  |  |  |  |
| 10. Did the statistical methods examine changes in outcome measures from before to after the intervention? Were statistical tests done that provided p values for the pre-to-post changes? | Yes |  |  |  |  |  |
|  | No | No | No | No | No | No |
|  | Other  (CD, NR, NA) * |  |  |  |  |  |
| 11. Were outcome measures of interest taken multiple times before the intervention and multiple times after the intervention (i.e., did they use an interrupted time-series design)? | Yes |  |  |  | Yes |  |
|  | No | No | No | No |  | No |
|  | Other  (CD, NR, NA) * |  |  |  |  |  |
| 12. If the intervention was conducted at a group level (e.g., a whole hospital, a community, etc.) did the statistical analysis consider the use of individual-level data to determine effects at the group level? | Yes |  |  |  |  |  |
|  | No |  |  |  |  |  |
|  | Other  (CD, NR, NA) * | NA | NA | NA | NA | NA |

**Table S4. Risk of bias for case report and case series**

| Study ID | | Boucoiran 2015 | Hoffer 2013 | Nóbrega 2013 | Hegazi 2013 | Hegazi 2012 | Taylor 2011 | McKeown 2010 | Pinnetti 2010 | Jaworsky 2010 | Trahan 2020 | Medeiros 2016 | Soh 2015 | Westling 2012 |
| --- | --- | --- | --- | --- | --- | --- | --- | --- | --- | --- | --- | --- | --- | --- |
| 1. Were patient’s demographic characteristics clearly described? | Yes  No  Unclear  Not applicable | Yes | Yes | Yes | Yes | Yes | Yes | Yes | Yes | Yes | Yes | Yes | Yes | Yes |
| 2. Was the patient’s history clearly described and presented as a timeline? | Yes  No  Unclear  Not applicable | No | No | No | Yes | No | No | No | Yes | No | Yes | No | Yes | No |
| 3. Was the current clinical condition of the patient on presentation clearly described? | Yes  No  Unclear  Not applicable | Yes | Yes | Yes | Yes | Yes | Yes | Yes | Yes | Yes | Yes | Yes | Yes | Yes |
| 4. Were diagnostic tests or assessment methods and the results clearly described? | Yes  No  Unclear  Not applicable | Yes | Yes | Yes | Yes | Yes | Yes | Yes | Yes | Yes | Yes | Yes | Yes | Yes |
| 5. Was the intervention(s) or treatment procedure(s) clearly described? | Yes  No  Unclear  Not applicable | Yes | Yes | Yes | Yes | Yes | Yes | Yes | Yes | Yes | Yes | Yes | Yes | Yes |
| 6. Was the post-intervention clinical condition clearly described? | Yes  No  Unclear  Not applicable | Yes | Yes | Yes | Yes | Yes | Yes | Yes | Yes | Yes | Yes | Yes | Yes | Yes |
| 7. Were adverse events (harms) or unanticipated events identified and described? | Yes  No  Unclear  Not applicable | Yes | Yes | Yes | No | Yes | No | Yes | Yes | Yes | No | Yes | No | Yes |
| 8. Does the case report provide takeaway lessons? | Yes  No  Unclear  Not applicable | Yes | Yes | Yes | Yes | Yes | Yes | Yes | Yes | Yes | Yes | Yes | No | Yes |
| Overall appraisal: Include Exclude; Seek further information | | include | include | include | include | include | include | include | include | include | include | include | include | include |

Note: Each item can be assessed as Yes, No, Unclear or Not applicable.


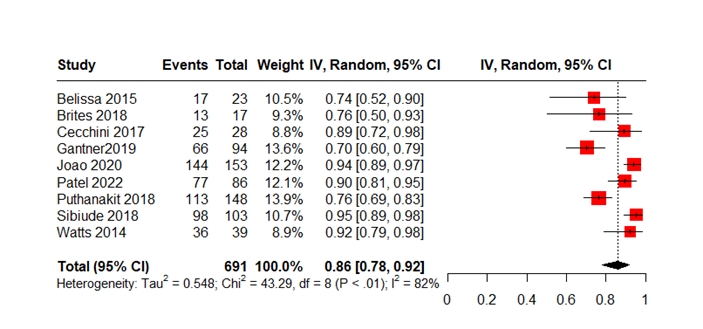


**Figure S1. Meta-analysis forest plot of virological suppression at delivery (defined as a viral load of ˂1000 copies/ml if RAL was initiated during the third trimester (≥28 weeks) or <200 copies/mL at delivery for those who initiate RAL before the third trimester (<28 weeks)**


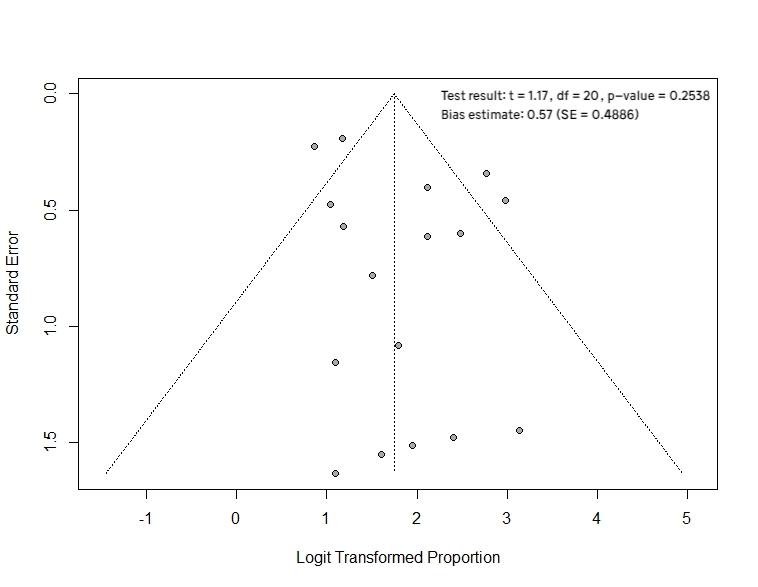


# Figure S2. Funnel plot of virological suppression at delivery (defined as a viral load of ˂1000 copies/ml if RAL was initiated during the third trimester (≥28 weeks) or <200 copies/mL at delivery for those who initiate RAL before the third trimester (<28 weeks)


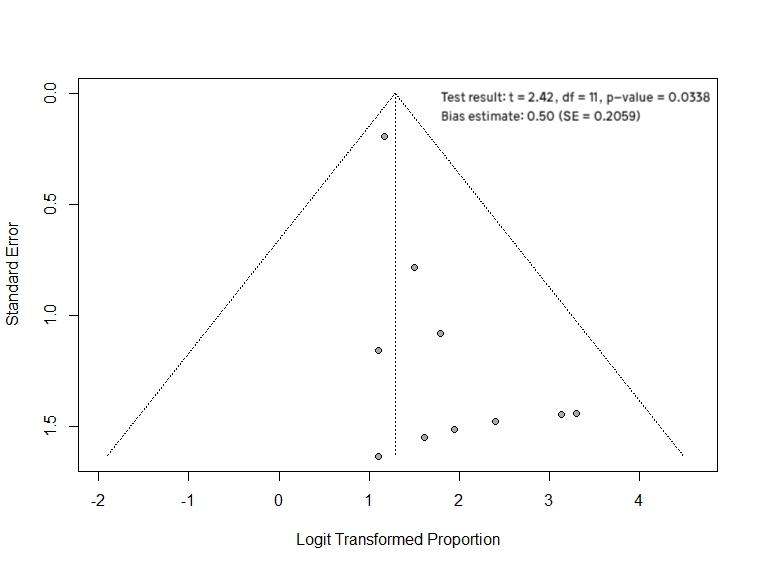


# Figure S3. Funnel plot of virological suppression (<1000 copies/mL) at delivery if RAL was used during the second trimester (≥ 14 weeks)
